# Workflow in Clinical Trial Sites & Its Association with Near Miss Events for Data Quality: Ethnographic, Workflow & Systems Simulation

**DOI:** 10.1371/journal.pone.0039671

**Published:** 2012-06-29

**Authors:** Elias Cesar Araujo de Carvalho, Adelia Portero Batilana, Wederson Claudino, Luiz Fernando Lima Reis, Rafael A. Schmerling, Jatin Shah, Ricardo Pietrobon

**Affiliations:** 1 Research on Research Group, Department of Surgery, Duke University, Durham, North Carolina, United States of America; 2 Cesumar, Universitary Center of Maringa, Paraná, Brazil; 3 UEM, State University of Maringa, Paraná, Brazil; 4 Oncologistas Associados Cancer Institute, Rio de Janeiro, Brazil; 5 Institute for Teaching and Research, Sirio Libanes Hospital, São Paulo, Brasil; 6 Research on Research Group, Duke-NUS Graduate Medical School, Singapore, Singapore; 7 Department of Surgery, Duke University Health System, Durham, North Carolina, United States of America; 8 Duke - NUS Graduate Medical School, Singapore, Singapore; 9 Research on Research Group, Duke University, Durham, North Carolina, United States of America; The George Washington University Medical Center, United States of America

## Abstract

**Background:**

With the exponential expansion of clinical trials conducted in (Brazil, Russia, India, and China) and VISTA (Vietnam, Indonesia, South Africa, Turkey, and Argentina) countries, corresponding gains in cost and enrolment efficiency quickly outpace the consonant metrics in traditional countries in North America and European Union. However, questions still remain regarding the quality of data being collected in these countries. We used ethnographic, mapping and computer simulation studies to identify/address areas of threat to near miss events for data quality in two cancer trial sites in Brazil.

**Methodology/Principal Findings:**

Two sites in Sao Paolo and Rio Janeiro were evaluated using ethnographic observations of workflow during subject enrolment and data collection. Emerging themes related to threats to near miss events for data quality were derived from observations. They were then transformed into workflows using UML-AD and modeled using System Dynamics. 139 tasks were observed and mapped through the ethnographic study. The UML-AD detected four major activities in the workflow evaluation of potential research subjects prior to signature of informed consent, visit to obtain subject́s informed consent, regular data collection sessions following study protocol and closure of study protocol for a given project. Field observations pointed to three major emerging themes: (a) lack of standardized process for data registration at source document, (b) multiplicity of data repositories and (c) scarcity of decision support systems at the point of research intervention. Simulation with policy model demonstrates a reduction of the rework problem.

**Conclusions/Significance:**

Patterns of threats to data quality at the two sites were similar to the threats reported in the literature for American sites. The clinical trial site managers need to reorganize staff workflow by using information technology more efficiently, establish new standard procedures and manage professionals to reduce near miss events and save time/cost. Clinical trial sponsors should improve relevant support systems.

## Introduction

While clinical trials have typically been conducted in developed countries such as the United States of America, developing countries have recently emerged as important new locations for clinical research [1]. In particular, the BRIC countries (Brazil, Russia, India and China) and VISTA (Vietnam, Indonesia, South Africa, Turkey, and Argentina), with their large and ethnically diverse populations, have become major players in the globalization of clinical research [2]. At the same time, pharmaceutical companies are differentiating clinical research capabilities across these countries to inform their investment decisions. Among the most important capabilities is the ability to deliver high quality data. As a result, efforts to improve and ensure the production of high quality data in all countries are essential for enhancing their competitiveness as viable locations for the conduct of clinical trials, especially in light of previous studies that have linked research in developing countries with lower levels of data quality [3].

Even usual errors in data acquisition and transcription during clinical trials can directly affect data quality, due to the additional effort necessary to correct possible errors and ensure an acceptable level of data quality. The typical example is that of a busy clinical research coordinator (CRC) who is not adequately trained to collect data and who passes the task of data entry to another CRC who might enter data that he/she does not understand.

While previous studies involving clinicians, radiology interpretation, healthcare management systems, have evaluated the relationship between workflow and data quality [4], [5], [6], [7], most have been conducted in a clinical rather than a research environment, frequently using workflow data that had been automatically captured. Although this approach is appealing, it does not apply to a clinical research setting where each step of a Principle Investigator (PI), Clinical research coordinator (CRC), nurse, receptionist and pharmacist is not automatically captured in an electronic system. Moreover, previous studies [5], [8], [9], [10], [11], [12] investigated methods of standardizing and improving the quality of clinical trial procedures. Between them, only a few workflow studies were conducted in research settings using a pure ethnographic approach [13].

Recent studies have used Unified Modeling Language (UML) [14] - a method that helps assess workplace efficiency, to assess and suggest improvements in clinical research workflow [15], [16], [17]. UML is composed of a set of graphical notations initially created to help understand software processes. It allows us to model a system and help visualize and understand its operation. It is currently used to model processes in diverse areas such as web applications, business processes and health care [14].

Despite their benefits, UML models are static and hence are limited by their description of a problem in a specific instance in time. Dynamic models can be simulated to understand their behaviour and analyze a range of “what if scenarios” thus providing crucial information before the practical implementation of a system level change/policy. Previous studies have [15], [18], [19], [20] used simulation techniques to represent diverse aspects of the real world in an interactive way. Systems Dynamics (SD) a modeling and simulation technique has been widely used to evaluate the behaviour of systems [21], [22], [23]. It includes a set of principles for modeling and simulation that are used to conceptualize and evaluate complex systems. Through a SD model, we can represent the relationship between elements, their activities as well as the flow and accumulation of information between them. We can also feed the model with qualitative and quantitative data and then create multiple models representing a variety of “what if” scenarios that help in decision making. Given the complex nature of health systems and clinical research, SD has previously been used to model decision support systems in this area [24], [25], [26], [27].

Although there is a wide variety of publications in each of these areas, to the best of our knowledge, no previous study has evaluated the workflow [28] of clinical trials in depth through a mix of techniques such as ethnographic studies to capture information about work routine, used UML [14] to graphically represent workflow in the form of activity diagrams [29] and SD modeling [4], [13], [30] to evaluate the resulting behaviour over time. Modeling and simulation performed before initiation of a clinical trial may help trial planners and managers to improve the quality of data and save time.

Accordingly, the objective of this study was to 1. Map the workflow of PIs, CRC’s, nurses, receptionists and pharmacists of two oncology clinical sites in Brazil, documenting ethnographic observations using a standard task diagram language [14] and 2. Introduce workflow modeling using UML and propose the use of simulation modeling to understand the underlying behaviour of the workflow model and 3. Conducting computer simulation experiments [31] using a system dynamics approach [21] to estimate work efficiency gains when solutions are applied.

## Methods

### Ethics

We obtained ethics approval from Comitê de Ética em Pesquisa do Hospital Pro Cardíaco, at Rua Dona Mariana, Rio de Janeiro, Brazil and Comitê de Ética em Pesquisa (CEPesq) of Hospital Sírio Libanês, São Paulo, Brazil for conducting the study at the trial sites in Rio de Janeiro and São Paulo respectively.

### Inclusion and Exclusion Criteria

We evaluated two oncology trial sites working with Qualidoc (consultant and training support at all levels of personnel in ISO9001: 2008) recommended by Agência Nacional de Vigilância Sanitária (ANVISA) - a National Agency of Sanitary Surveillance in Brazil. Both sites were located in Brazil: the first being a clinic-based environment in the city of Rio de Janeiro with one PI oncologist, two nurses CRCs, one pharmacist, one clinical research administrator and fourteen studies in phase III and the second being a hospital-based environment in São Paulo, with two PIs oncologists, two nurses CRCs, four pharmacists, one clinical research administrator and three studies in phase III. Both sites present a high volume of trial recruitment. We considered sites with an active phase III or IV trials as high volume sites. We excluded Phase I and II trials since they do not represent the majority of trials conducted in BRIC countries, also presenting a very diverse workflow when compared to phase III/IV trials.

### Operational Definitions

Although some technical terminology might encounter different definitions across clinical trials, workflow, and qualitative research literature, for the purposes of this manuscript, we have standardized the most common concepts to facilitate understanding (Table 1).

**Table 1:** Terminology standardization.

Term	Description
Protocol	Clinical trial documentation that outlines the objective, methodology, design, analysis plan. It also describesthe background and reason for the conduct of the study and puts forth a standard method forthe conduct of the clinical trial [22]
Actor	A person who is a part of the trial workflow and interacts with tasks or other actors [21]
Principal Investigator (PI)	An individual who is directly responsible for the conduct and completion of a funded clinical trial.He/She directs the research project and reports study results directly to the sponsors [22]
Sub-Investigator	Physicians designated and supervised by the PI to perform procedures and monitor subjects in the clinicaltrial [22]
CRC (Clinical Research Coordinator)	Individuals responsible for operational tasks and for providing support to PI/Subinvestigator in a clinical trialstudy. They could be involved in the process of inclusion, recruitment and still maintain the registry ofparticipants, provide the signature of informed consent forms and schedule procedures and lab tests,ensuring accuracy of source documentation, dispensing study medications and maintaining databases withclinical research data, filling the CRF [22]
Research Subject	Once the patient agrees to be a part of a clinical trial and signed the informed consent document,he/she becomes a research subject [22]
CRF (Case Report Form)	It is a paper-based or electronic record of subject data specifically used in clinical trial research [22]
Source Document	a document where collected data is first recorded for a clinical trial and later entered in the CRF [22]

### Ethnographic Study

Two researchers from our team [AB, EC] made ethnographic field observations for approximately 40 hours and conducted qualitative interviews at two sites in Rio de Janeiro and São Paulo (Brazil), where they shadowed PIs, CRCs, nurses, receptionists and pharmacists. We recorded information on tasks (S1), interactions between other actors (defined in table 1), communication patterns, information needs, and other aspects related to their workflow. We conducted semi-structured interviews using open-ended questions to complement and fill gaps in our ethnographic observations, being broadly directed to (1) understand the average order of tasks in different stages of each protocol, (2) main causes that affect the average order of tasks, including the most common interruptions; (3) main communication contacts for each actor, how frequently communication occurs, how frequently the workflow is affected by the patterns of communication and what type of noise affects the performance of communication flow; (4) documentation models and migration from paper to computer interface used for documentation; (5) How documentation templates are affected when trial site staff are confronted with any query about events, for example, a subject enrolled sometime ago; (6) information necessary that affect the workflow, causing the CRC to search out information from different sources and need to contact other personnel; (7) How clinical trial workflow has been integrated with information technology and if there are in any area where some disconnection between them exists causing a disruption in the regular workflow**.** These seven concepts were extracted from the study of Khan et. al. in [13].

Observations were made in a non-participatory manner, in that observers were not directly involved in actively collecting data for the clinical trial, therefore attempting to minimize the Hawthorne effect that is characteristic of every observational study [32]. Observers did not have access to protected health information (PHI) or any specific data related to the ongoing clinical trials to ensure confidentiality. Since observers did not have access to raw data for privacy and security reasons, the identification of data quality issues was made following the concept of near miss events (the happening of an unexpected event that did not generate important problems, but had big potential to) [33]. Following is an example of a near miss event: a CRC schedules an appointment in his/her personal electronic calendar, notifying other staff personnel about the same. On the day of appointment, in the event of the CRC’s absence, the patient would still be received and assisted by other staff personnel at the site. Yet some important detail/document, available only to the CRC may be missed during this process. Consequently, it may generate a greater problem at the end of the study.

When we observed a task that had a higher potential of near miss events, we highlighted it as a potential problem. Information on tasks was later submitted back to the staff at each of the two sites to ensure that information was not misrepresented, thus ensuring appropriate data triangulation [34].

All data collected through the ethnographic study and interviews were transcribed in a Google Docs Word file. After analysis, we synthesized emerging themes using atlas.ti [35], a software for qualitative data analysis. Each interview was coded by two researchers using Grounded Theory methods [36] where emerging themes were extracted from patterns evolving from the text and by avoiding pre-conceived hypothesis. Ambiguities if any were resolved by discussion. Categories were reduced to major themes through discussion amongst the study team while re-reading the transcripts.

### Observation Categories

Observers documented CRC tasks by selecting tasks descriptions from a list of tasks compiled from the emerging themes obtained from the previously described ethnographic study and by findings from previous studies [13]. A large group of major and minor categories were then used to facilitate the classification of any possible CRC tasks. For the purpose of analysis, this large group of tasks was summarized into a smaller number of categories.

### Workflow Mapping

Information from the ethnographic study was condensed using a modified version of UML (Unified Modeling Language) Activity Diagrams (UML-AD), version 2.0 [37]. All modeling was conducted using Astah community version 6.1 [38]. The variation of activities between two sites was represented by the workflow (S6) pattern “exclusive choice” [39]. Exclusive choice represents a situation where there are two or more exclusive alternate paths to a workflow and only one of them can be chosen. The terminology for workflow and UML- AD diagram are present in Supporting information S6.

### Preliminary Simulation Model

Next, we formed an external panel with five clinical researchers, affiliated to the Research on Research group (RoR), Duke University, to discuss the findings and summarize it as a simulation model. The simulation was performed by taking the UML activity diagram as the basis for a System Dynamics model [21], [22], [23]. All modeling was conducted using Vensim DSS [40]. This process involved two steps. First, a baseline model was created to reproduce the current workflow of each clinical trial site along with the reported problems related to near miss events for data quality (Table 2). A system analysis of each major loop in the system identified points where workflow could be potentially improved in terms of a better execution of tasks. Second, the model was presented to each participant for feedback, who suggested modifications. Interventions were then tested in subsequent models, where we simulated an improvement in the system. Finally, the model was fed with qualitative data and simulated using different scenarios.

**Table 2:** Variables classified as problematic on visited sites.

**Visit-EDC asynchrony**	The CRC does not have the time to transcribe the content from the paper-based or electronic medical record to the computerized electronic data capture (EDC) in atimely manner.
**EMR-EDC non-interoperability**	Electronic medical record (EMR) not integrated with EDC, therefore may cause error orlack of information during transcription from one the EMR computer screento the one for the EDC.
**CRC-data entry discontinuity**	At the time of the encounter with the subject, investigator takes notes on the EMR,nurse takes note on nurse plan and CRC makes the final data entry into EDC.
**Lab-EDC non-interoperability**	Laboratory system not integrated with EMR, requiring re-entry with potential for transcription errors.
**Decentralized accounting for drug dispensation**	System for drug stock control between pharmacy and trials use different systems,with a potential for discrepancy and error.
**Lack of decision support systems to assist CRC**	CRC frequently has to consult a variety of documents not available at the time of interaction with research subject. This factor is compounded by a single CRC beingin charge of multiple studies supported with paper-based documentation, whichmight lead to confusion and error.

## Results

Overall, the two sites evaluated in this study demonstrated consistent data collection system, with only a few discrepancies between them. The inconsistencies were related to specific infrastructure aspects. In addition, a group of areas with higher potential for flow quality issues, i.e., following the near-miss event concept, were also identified, mapped and simulated. In total, we observed the activities of four Clinical Research Co-ordinators (CRCs), and identified a total of 139 tasks (Refer to supporting information S1).

### Ethnographic Field Observations and Workflow Mapping using UML Activity Diagrams

#### General observations

The UML-AD detected four major activities in the workflow, namely (a) evaluation of potential research subjects prior to signature of informed consent (S2), (b) visit to obtain subject́s informed consent (S3), (c) regular data collection sessions following study protocol (S4), and (d) closure of study protocol for a given project.

Activity A, prior to the signature of the informed consent, the potential research subject underwent a clinical exam as well as additional laboratory exams as per clinical site routine. These are common pathological exams and work as a pre-screening method. The results of these exams help in the evaluation of inclusion and exclusion criteria. During activity B - the visit to obtain subject́s informed consent, potential subjects received an explanation about the entire study, described in the informed consent form while being offered the opportunity to ask questions. The PI then requested the subject to sign the informed consent. At this point an individual schedule for the study subject was established. Activity involved a series of encounters representing regular data collection sessions following the variations across different study protocols. Finally, during activity D, the subject either reached completion or was excluded from the study protocol, when study closure was achieved, including the collection of any remaining interventional drug when applicable and also included the plan for any further follow-up when required by protocol. Since each of these activities is distinct, they were represented by different UML-ADs (Refer Supporting information S2, S3, S4).

#### Observations of workflow tasks with higher likelihood of quality issues

Our field observations pointed to three major emerging themes (based on table 1) corresponding to areas where workflow was associated with potential near miss events for data quality issues.

#### Lack of standardized process for data registration at source document

The first emerging theme resulted from the presence of a number of intermediate documents in the transfer of data from research subject to the electronic CRF by CRC. Examples include: (a) Nurse reports were paper-based and later manually transferred to an electronic data capture system; b) Drug stock control was made on tables of text editor or spreadsheet and then printed; c) Results from laboratory exams were printed from their home pages or received by email and subsequently printed, or printed from laboratory computer system applications. Although all of these sources do not violate any current Good Clinical Practice regulations, as CRFs were filled out based on the original source data, the presence of intermediate documents – even if used only for care purposes - increases the odds of error secondary to unintentional transcription errors. In addition, these intermediate steps represent rework.

#### Multiplicity of data repositories

We observed that the information being transferred was registered in different binary files such as Microsoft Word and Excel files. The files were not linked and later were manually transferred using a copy and paste functionality. In one of the sites, documents directly filled out with subject́s information were not directly inserted into the CRF by the CRC, but remained in paper-format (source document) for later transcription into the electronic CRF.

The paper-based documentation trail also results in searchability problems, in that, locating specific data fields for validating systems in the area or when checking for possible errors was time-consuming. Adding to the complexity of this process, research nurses were required to fill out multiple forms in accordance to study protocol, this information being transcribed by investigators and later copied and finally pasted by the CRCs. Other parallel systems also contained different, non inter-operable systems such as pharmacy services using multiple Microsoft Excel and Word documents for stock control, drug validity, and medication disposal.

#### Scarcity of decision support systems at the point of research intervention

Given that a single CRC is concomitantly in charge of multiple studies, it is not unusual that the information about specific details pertaining to each study were sometimes forgotten. This oversight resulted in a major rework loop, in that the correction could only be accomplished through the central coordinating center located in the European Union, not before having to contact the central coordinator for Brazil. The total rework process resulted in a total time loss of approximately two uninterrupted hours. In another instance, the complete period to complete a CRF lasted about one hour, reportedly at least three times more than what that task would usually require. According to the CRC, the process was slow since, although she had undergone previous training, that training had occurred several months before and most study-related processes were new, requiring constantly re-checking to ensure information accuracy. Since the documentation was long and not easy to search, the whole workflow was significantly delayed, ultimately affecting the CRC’s ability to complete it on time. With this delay, some of the CRF completion was left to another person on staff, enhancing the probability of near miss events for data quality issues. Other unexpected tasks for which there is no automation also prevented that the CRC complete the protocol on time, such as accounting issues related to the appointment such as the CRC spending one complete morning solving financial problems related to the study. In addition, study manuals frequently were confusing and lacked appropriate usability as attested by confusion generated while the CRC attempted to interpret them.

### Preliminary Simulation Model

**Figure 1:**
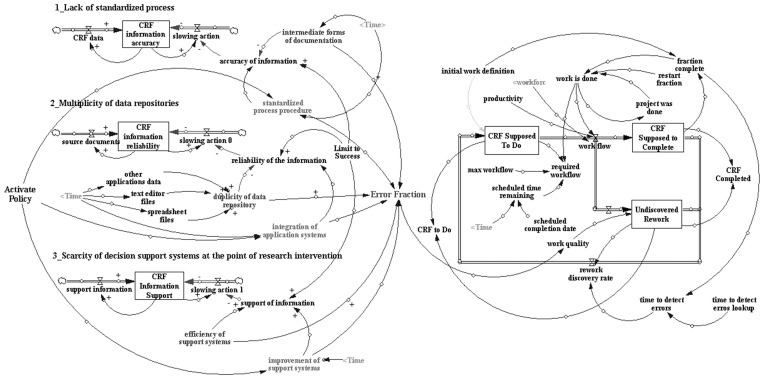
System dynamics model showing the three major emerging themes identified and rework caused by these themes. Available for best visualization in http://goo.gl/XydTd.

The preliminary simulation model (Figure 1) envisioned the following three main emerging themes (S5) generating rework: 1) Lack of standardized process for data registration at source document, 2) Multiplicity of data repositories and 3) Scarcity of decision support systems at the point of research intervention. The model has the following features: (a) all emergent themes are represented as stocks (rectangle), (b) a blue arrow represents information input and (c) a red arrow represents some action that will limit the growth of a stock.

The theme “lack of standardized processes” has as its central feature represented by a stock “CRF information accuracy”. Aligned with our ethnographic findings, the variable representing “intermediate forms of documentation” has a negative influence over “accuracy of information”. The variable “accuracy of information” will speed up the processing of CRF completion. In other words, the greater the “accuracy information”, less delay in successfully completing CRFs we will have.

The second theme “multiplicity of data repositories” is represented by a stock of reliable CRF information. Aligned with our ethnographic observations, it was seen in the form of three types of data repositories of information that would lead to the same information in different formats (Word documents, spreadsheets in Excel and software applications like central pharmacy or laboratories). In our model, we simulated how data multiplicity would affect reliability of CRF information. In our model the variable “data repository multiplicity” is influenced by three distinct variables: “other applications” (laboratories application, central pharmacy application and others), “text editor files” (Word documents), and “spreadsheet files” (Excel documents). The variable “data repository multiplicity” has a negative effect on the variable “information reliability.” In other words, the greater the multiplicity of data sources, the less reliable the information and the greater the delay in successful completion of CRFs.

The third theme “scarcity of decision support system at the point of research intervention” indicates that the current workflows for filling out paper and computer-based CRFs are in need of a better decision support system. The model also contains a variable representing the “efficiency of a support system,” which positively impacts the speed in completing CRFs.

Additionally, we can observe that the main variables of our model “intermediate forms of documentation”, “data repository multiplicity” and “support of information” are influencing another variable called “Error Fraction”. The variable “Error Fraction” is used in our model to represent the stocks – ‘CRF to Do’, ‘Rework’ and ‘CRF Completed’. It receives information from the left part of model, representing the three emergent themes and has a directly proportional relationship with the variable - “Rework” flow. This means that higher instances of ‘Error’ will generate more rework and as a result lesser CRFs will be completed.

As we did not have access to real data, we made the following assumptions: the values for “intermediate of documents” and “multiplicity of data repositories” was simulated starting on zero and growing each six months until the limit of 20% and 30% respectively (Refer to supporting information S5). We did this to simulate a situation where the staff started working with no problems but faced them increasingly over time. The value of efficiency of support system were simulated with 50% all the time, with this parameter we simulate a system with 50 percent deficiency.

**Figure 2:**
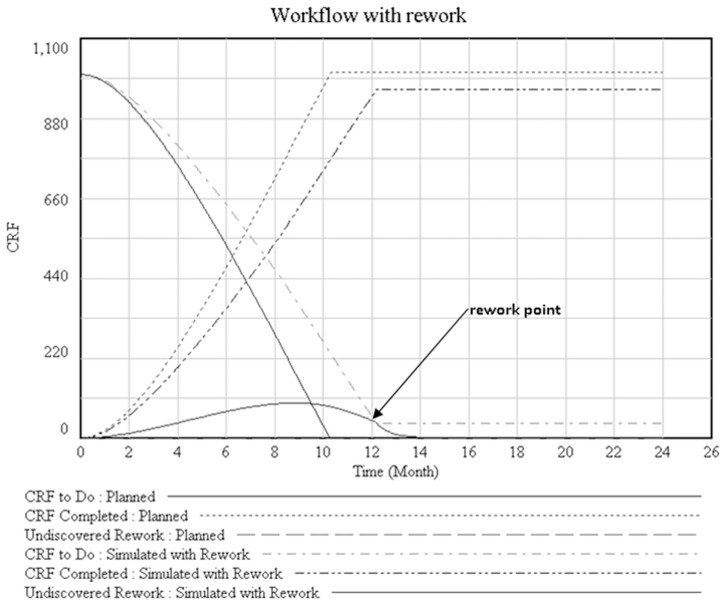
CRF’s to do and CRF’s completed in two situations: planned and simulated with rework.

We present two situations based on the outcomes of simulating the model (figure 2). In the first situation labeled as “planned”, we assume the lack of an error in the work done. The simulation results represent CRFs to be done and CRF́s completed. In the second situation labeled as “Simulated with Rework” we attempted to simulate of what happened on observed environment. This simulation was based on three emergent themes presented in this manuscript. In this second situation work could not be completed as planned. This was primarily a consequence of rework that was generated because of error fraction caused by emergent themes consequences (rework point on figure 2).

### Policy Model Results

**Figure 3:**
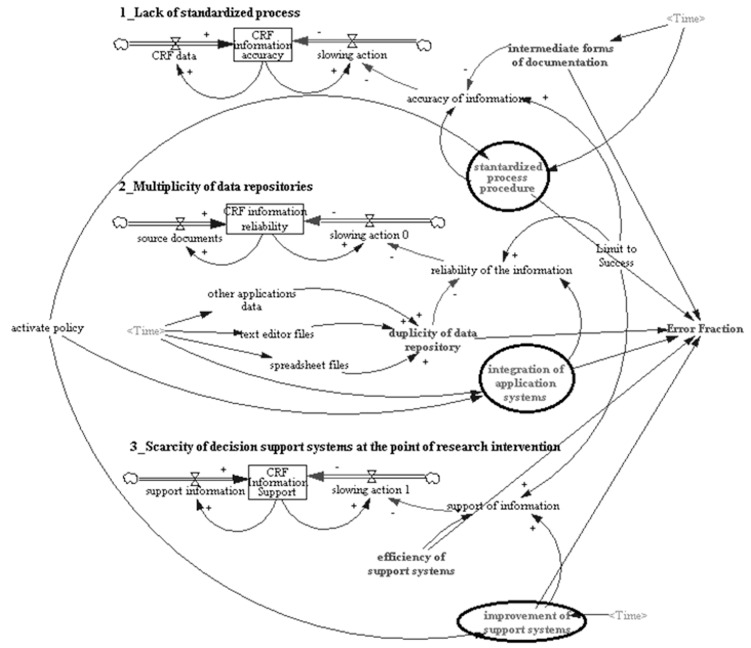
Dynamic model with the policies suggested (circled). Available for best visualization in http://goo.gl/XydTd.

The policy model results were divided into three categories of policies with an aim to reduce the rework: (a) Standardized process procedure, (b) Integration of application systems, (c) Improvement of support systems (figure 3). In “standardized process procedure” policy, we suggest the clinical trial administrative staff of each site to periodically revise their standard procedures and perform training with the research team when working with CRFs to decrease the creation of intermediate documents. For the policy “integration of application systems” the clinical trial administrative staff must work together with IT staff of a site and analyse all the data repositories created in duplicity (as observed during our ethnographic study) and create a solution where the clinical trial staff will insert information in only one application to avoid these duplicities. Finally, in the “improvement of support systems” policy, the clinical trial administrative staff and sponsors need to work more efficiently to obtain support information more fast and friendly when working with CRF. For each one of these policies the PI, CRC’s, nurses, pharmacists and receptionist involved in clinical trial procedures must be trained in new methods and procedures that can facilitate the use of information and resources more efficiently while reducing rework.

**Figure 4:**
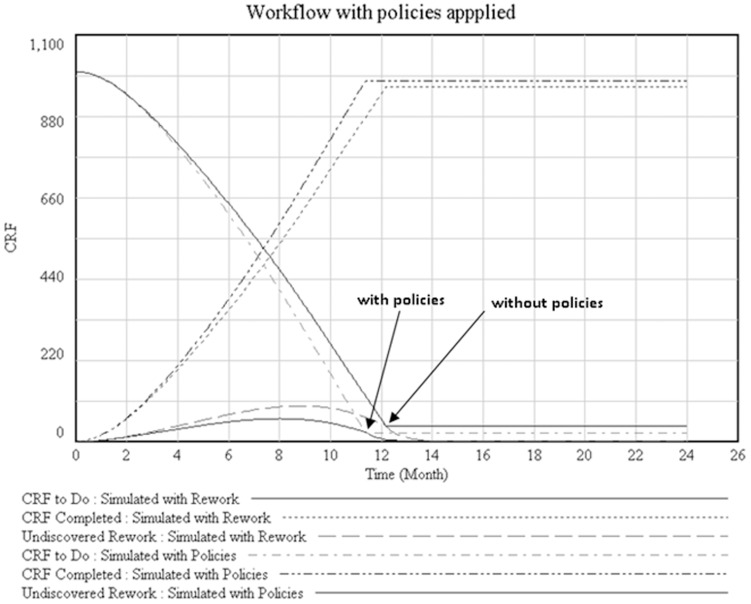
CRF’s to do and CRF’s completed in two situations: simulated with rework and simulated with policies suggested.

As a result of policies applied (figure 4), we observe the latter leads to more CRF’s completed, resulting in less rework. Rework does not disappear altogether but reduces. In course of time, if policies are well implemented, rework should tend towards zero.

**Figure 5:**
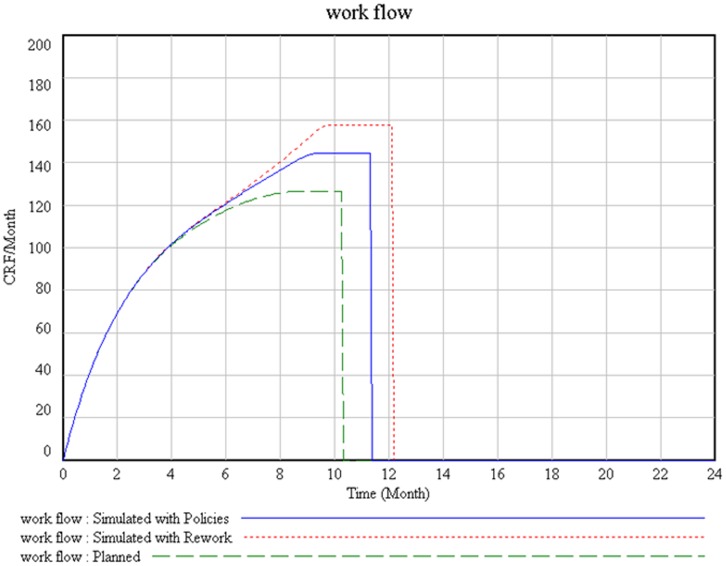
The Workfow of all process simulated for three situations: Planned, with reword identified and wirh policies suggested.

Figure 5 gives us an idea of workflow behaviour in our simulation. The dotted line represents workflow planned, which should be complete a thousand of CRFs for ten months. The straight line represents the simulation performed with rework caused by three emerging themes identified. In this situation, the workflow has been increased in order to be able to complete a thousand CRFs which will need twelve months. The dotted line represents workflow with the implementation of policies where the time to complete CRF was reduced. The ideal workflow may depend on factors that of not immediate implementation, therefore the implementation of the policies had a small reduction of rework and still kept it.

## Discussion

To the best of our knowledge, this is the first study evaluating and mapping workflow at clinical trial sites while also conducting a simulation to represent the potential for improvement once the main issues were addressed.

In our study, several factors affecting workflow pointed towards the lack of interoperability between the electronic medical record and electronic data capture system at the clinical trial site, namely visit-EDC asynchrony, EMR-EDC non-interoperability, lab-EDC non-interoperability, decentralized accounting for drug dispensation). Factors affecting this relation included the presence of standalone and disconnected systems taking care of schedule management, administration, lab reporting, medical records, and research databases. This results in fragmented and siloed data that are seldom useful for research purposes. In many cases, it has been noted that the data from EMR cannot be directly utilized in a clinical trial environment on account of incompatible data formats and non adherence to clinical research regulations [41]. As a consequence, despite the availability of state of art systems, information is frequently recorded manually on charts followed by separate data entry into the EMR and EDC. The lack of interoperability thus leads to less efficiency, duplication [41], and errors, leading to disruption of the clinical trial pipeline and damages estimated in million dollars/year.

In our study, near miss events for data quality were also influenced by a discontinuity in performance of a specific task. Specifically, rather than having one individual performing the entire task, the process was broken and implemented by more than one individual. Although this practice is aimed at workflow distribution and enhancing productivity and efficiency of different individuals it also serves to increase the possible flow quality problems resulting from multiple individuals with different levels of information about the subject handling the data. Previous literature suggests that interruption during task implementation may not only have a negative effect on task performance but also lead to serious errors [42]. For example, CRCs may receive unexpected workload and thus forget a critical portion of their task lists or incur in data entry errors [13].

We also noted that an absence of decision support systems for clinical research coordinators had a significant influence on their task performance and resultant near miss events for data quality. Clinical research coordinators and physicians frequently need to answer queries or consult the study material while evaluating a research subject. Lack of readily available guidelines has implications on time and effective subject management in a clinical trial. In our study we identified difficulties encountered by CRC to quickly locate information while handling long paper-based protocols. This difficulty could be alleviated with, for example, electronic documents with better navigability that could be immediately queried at the time when they are needed. Although the benefits of decision support systems in enhancing the safety and quality of clinical practice [43], [44], [45], reducing errors [46], [47] and ensuring adherence to practice guidelines [48], [49] has amply been demonstrated in the past, they are equally useful in clinical research settings. In the latter context, decision support tools have been effectively used to determine subject eligibility [50], [51]. Based on our results, the implementation of a decision support tool to guide CRC tasks would similarly result in the optimization of clinical trial tasks.

Although our report has made a significant contribution in terms of understanding, representing, and simulating strategic workflow decisions to reduce the incidence of near miss data quality issues in clinical trial sites, our study has limitations. As in any observational study, the presence of observers in the clinical trial site might interfere with the way subjects behave, potentially introducing bias into our observations [52]. However, since most of our findings were confirmed by triangulation [53] to be part of the daily routine of the investigated sites, this effect should be considered minimal.

The results obtained from the suggested interventions implemented in our model simulation were not validated through real life implementation, since it would require a substantial investment from the site and sponsor’s perspective. Given the highly regulated environment within clinical trials, the level of variability in our UML model is reduced thus enhancing its applicability. Several of the investigators were familiar with the clinical trial process and, therefore, could have unintentionally introduced their opinions into the observational process.

Another issue in comparing workflows across trial sites is the absence of a taxonomy listing variables that should be compared. This taxonomy would ideally focus on areas of dissonance between workflow and data quality and requires a specific article describing this approach. Moreover, although the two sites where we collected data represent active Cancer trial sites in Brazil, our sample size was small and may not be judged representative. A larger sample size could increase the generalizability of our results, but qualitative studies are aimed at depth rather than generalizability, the former being achieved by the data collected at each site.

Although we conducted our study in a developing country (Brasil), there is little evidence to suggest that the problems we describe are restricted to developing countries. Moreover, the low granularity of our system level recommendations is justified given its occurrence every time a new approach is applied to a field. Given that, to the best of our knowledge, this is the first study evaluating and mapping workflow at clinical trial sites while also simulating its results using System Dynamics.

Finally, we make a substantial assumption that near-miss events are a proxy for real data quality events. This assumption was made given the qualitative nature of our study, where samples are frequently small and exploratory, therefore unable to reach results that would qualify as definitive. However, we would also like to point that an assumption of association between near miss and “real events” has been made in a wide range of the literature and ratified by entities such as the Institute of Medicine in its report “To err is human” [54].

In conclusion, in order to reduce rework and optimize trial performance, it is essential to improve the efficiency prevailing in trial sites. Clinical trial sites and sponsors should review and improve their standard procedures and manage human resource allocation to better address the workflow issues summarized in our study. Sponsors and site managers can improve support systems for CRCs. Although some interventions such as integration between EMR and EDC, might not seem cost-effective or even technologically possible at this time, strategic decisions should be made to facilitate their future integration. For example, when planning for an EMR system, hospital administrators should evaluate the ease of integration with external EDCs. Also, future research should integrate systems modeling simulation so that these changes can be constantly monitored and frequently evaluated in the quest for data quality excellence. As a future study we intend to submit a new manuscript about the new taxonomy for what should be compared in workflows across sites focusing on areas of dissonance between workflow and data quality.

## Supporting Information

Supporting Information S1 **List of all task categories found through the field notes.**
(DOC)

Supporting Information S2 **UML - Activity Diagram for pre-signature consent form process.**
(DOC)

Supporting Information S3 **UML - Activity Diagram for signature consent form and first visit.**
(DOC)

Supporting Information S4 **UML - Activity Diagram for next visits.**
(DOC)

Supporting Information S5 **Emergent themes main variables in Vensim simulation software.**
(DOC)

Supporting Information S6 **Workflow Terminology.**
(DOC)
