# Supplementary material for: Workflow in Clinical Trial Sites & Its Association with Near Miss Events for Data Quality: Ethnographic, Workflow & Systems Simulation
Source: PLoS One. 2012 Jun 29;7(6):e39671. doi: 10.1371/journal.pone.0039671 (PMC3387261; doi:10.1371/journal.pone.0039671)
Supplement: Supporting Information S1 — List of all task categories found through the field notes. (DOC) [file pone.0039671.s001.doc]

**S**1 - List of all task categories found through the field notes

| **Seq** | **Actor** | **Theme** | **Category** | **Task** |
| --- | --- | --- | --- | --- |
| 1 | PI | Lack of Standardized Process  Multiplicity of data repository  Scarcity of decision support systems | Analysis of Potential Subject | Obtain a history of present illness, medical history, family medical history, social and personal information and enter it into electronic medical record |
| 2 | PI | CT Routine | Analysis of Potential Subject | Analysis of Potential Subject of Perform a physical exam and enter the results in electronic medical record |
| 3 | PI | CT Routine | Analysis of Potential Subject | Inform potential research subject that he is not eligible to be participating in the study |
| 4 | PI | Lack of Standardized Process | Analysis of Potential Subject | Request exams on the possible research subjects by electronic medical record |
| 5 | PI | Lack of Standardized Process | Analysis of Potential Subject | Adjourn encounter with potential research subject and clinical coordinator |
| 6 | PI | Lack of Standardized Process | Analysis of Potential Subject | Print guides on exam requests |
| 7 | PI | CT Routine | Analysis of Potential Subject | Give the guides on exam requests to possible research subjects |
| 8 | PI | Lack of Standardized Process | Analysis of Potential Subject | Adjourn encounter with potential research subject and clinical coordinator |
| 9 | PI | Lack of Standardized Process | Analysis of Potential Subject | Analyze/confirm exam results in electronic medical record |
| 10 | PI | CT Routine | Analysis of Potential Subject | Communicate to clinic research team whether or not the potential research subject is eligible to participate in the clinical trial |
| 11 | PI's Administrative Assistant | CT Routine | Analysis of Potential Subject | Bring possible research subject and research coordinator to PI´s office |
| 12 | PI's Administrative Assistant | Lack of Standardized Process | Analysis of Potential Subject | Gather information on potential research subject and enter it into electronic medical record |
| 13 | PI's Assistant | CT Routine | Analysis of Potential Subject | Introduce potential research subject and research coordinator |
| 14 | PI's Assistant | CT Routine | Analysis of Potential Subject | Lead potential research subject and research coordinator to PI´s office |
| 15 | Research Coordinator | CT Routine | Analysis of Potential Subject | Accompany potential research subject to PI´s office |
| 16 | Research Coordinator | CT Routine | Analysis of Potential Subject | Greet the potential research subject |
| 17 | Research Coordinator |  | Analysis of Potential Subject | Allow potential research subject to return home |
| 18 | Research Coordinator | CT Routine | Analysis of Potential Subject | Communicate exams appointment date to potential research subject |
| 19 | Research Coordinator | CT Routine | Analysis of Potential Subject | Determine if potential research subject is willing to participate in the clinical trial |
| 20 | PI | CT Routine | Clinical Trial Routine | Obtain a medical history, check vital signs and identify chief complaints |
| 21 | PI | CT Routine | Clinical Trial Routine | Perform a physical exam |
| 22 | PI | CT Routine | Clinical Trial Routine | Analyze exam results and determine if research subject has progressed |
| 23 | PI | Lack of Standardized Process | Clinical Trial Routine | Consult exams results previously entered in the medical record by the research coordinator |
| 24 | PI | CT Routine | Clinical Trial Routine | Release research coordinator and research subject |
| 25 | PI's Administrative Assistant | CT Routine | Clinical Trial Routine | Greet research coordinator and research subject |
| 26 | PI's Administrative Assistant | CT Routine | Clinical Trial Routine | Notify PI that research coordinator and research subject have arrived |
| 27 | PI's Administrative Assistant | CT Routine | Clinical Trial Routine | Direct research coordinator and research subject to PI´s office |
| 28 | Research Coordinator | CT Routine | Clinical Trial Routine | Screen research subject per protocol to verify inclusion/exclusion criteria |
| 29 | Research Coordinator | CT Routine | Clinical Trial Routine | Send research subject to nurse |
| 30 | Research Coordinator | CT Routine | Clinical Trial Routine | Give research subject a diary and explain how it should be completed |
| 31 | Research Coordinator | Lack of Standardized Process | Clinical Trial Routine | Record research subject's appointment date and time in Microsoft Outlook calendar |
| 32 | Research Coordinator | Lack of Standardized Process | Clinical Trial Routine | Record research subject's appointment date and time in shared calendar |
| 33 | Research Coordinator | Lack of Standardized Process | Clinical Trial Routine | File nursing plan form |
| 34 | Research Coordinator | Multiplicity of data repository | Clinical Trial Routine | Update medication stock count in Microsoft Excel |
| 35 | Research Coordinator | CT Routine | Clinical Trial Routine | Direct research subject and printed results to PI |
| 36 | Research Coordinator | CT Routine | Clinical Trial Routine | Collect research subject's diary |
| 37 | Research Coordinator | Lack of Standardized Process | Clinical Trial Routine | Schedule a new appointment for research subject to return and communicate appointment time to him/her |
| 38 | Research Coordinator | CT Routine | Clinical Trial Routine | Give research subject a new diary |
| 39 | Research Coordinator | Lack of Standardized Process | Clinical Trial Routine | Adjourn encounter with research subject |
| 40 | Research Coordinator | Lack of Standardized Process | Clinical Trial Routine | Record research subject´s appointment date and time in Microsoft Outlook calendar |
| 41 | Research Coordinator | Lack of Standardized Process | Clinical Trial Routine | Record research subject´s appointment date and time in shared calendar |
| 42 | Research Coordinator | CT Routine | Clinical Trial Routine | Collect remaining drug from research subject (if it was not infused or injected) |
| 43 | Research Coordinator | CT Routine | Clinical Trial Routine | Adjourn encounter with research subject |
| 44 | Research Coordinator | Multiplicity of data repository | Clinical Trial Routine | Open drug inventory control in Microsoft Excel |
| 45 | Research Coordinator |  | Clinical Trial Routine | Verify prescribed dose in EMR computer-based |
| 46 | Research Coordinator | Multiplicity of data repository | Clinical Trial Routine | Verify that returned drugs match information in drug inventory control spreadsheet |
| 47 | Research Coordinator | CT Routine | Clinical Trial Routine | Call research subject and ask what happened |
| 48 | Research Coordinator | Scarcity of decision support systems | Clinical Trial Routine | Consult protocol on procedures for missing drugd |
| 49 | Research Coordinator | Scarcity of decision support systems | Clinical Trial Routine | Follow protocol's procedures for missing drugs |
| 50 | PI | CT Routine | Consent Form Signature | Present and explain consent form to potential research subject |
| 51 | PI | CT Routine | Consent Form Signature | Adjourn meeting with research subject |
| 52 | PI | CT Routine | Consent Form Signature | Ask research subject to sign consent form |
| 53 | PI | CT Routine | Consent Form Signature | Deliver two copies of signed consent form to research coordinator |
| 54 | PI | CT Routine | Consent Form Signature | Adjourn encounter with potential research subject |
| 55 | Research Coordinator | CT Routine | Consent Form Signature | Meet potential research subject |
| 56 | Research Coordinator | CT Routine | Consent Form Signature | Show potential research subject to the PI´s office |
| 57 | Research Coordinator | CT Routine | Consent Form Signature | Adjourn encounter with potential research subject |
| 58 | Research Coordinator | CT Routine | Consent Form Signature | After a number of days specified by the protocol, call the potential research subject and request his/her participation and signed consent form |
| 59 | Research Coordinator | CT Routine | Consent Form Signature | Give research subject a copy of consent form |
| 60 | Research Coordinator | CT Routine | Consent Form Signature | File other consent form |
| 61 | Research Coordinator | Scarcity of decision support systems | CRF Routine | Consult CRF manual |
| 62 | Research Coordinator |  | CRF Routine | Register research subject information in CRF |
| 63 | Research Coordinator | Lack of Standardized | CRF Routine | Update CRF with information on nursing plans and procedures |
| 64 | Research Coordinator | Lack of Standardized | CRF Routine | Enter exam results in CRF chart |
| 65 | Research Coordinator |  | CRF Routine | Enter data from diary into CRF |
| 66 | Research Coordinator | Lack of Standardized | CRF Routine | Enter data from nursing plan into CRF |
| 67 | PI | CT Routine | EMR Routine | In the electronic medical chart, record whether or not the potential research subject is eligible to participate in the study |
| 68 | PI | CT Routine | EMR Routine | Access medical records and enter information about: physical exam, vital signs, conduct, current therapies, concomitant medication and research subject´s progress |
| 69 | PI | Lack of Standardized | EMR Routine | Enter nursing plan in electronic medical record |
| 70 | Research Coordinator | Lack of Standardized | EMR Routine | Record exam results in electroni medical record |
| 71 | Pharmacy | CT Routine | Farmacy Activities | Answer nurse´s phone call |
| 72 | Pharmacy |  | Farmacy Activities | Draw up drug |
| 73 | Pharmacy | Multiplicity of data repository | Farmacy Activities | Record drug release into pharmacy's information system |
| 74 | Pharmacy | CT Routine | Farmacy Activities | Send drug to nurse |
| 75 | Clinical Analysis | CT Routine | Laboratory Exams | Analyze collected specimen |
| 76 | Clinical Analysis | Lack of Standardized Process | Laboratory Exams | Record pathology report in laboratory information system |
| 77 | Clinical Analyst or Pathologist | CT Routine | Laboratory Exams | Analyze collected specimen |
| 78 | Clinical Analyst or Pathologist | Lack of Standardized Process | Laboratory Exams | Record pathology report in laboratory information system |
| 79 | Laboratory | Lack of Standardized Process | Laboratory Exams | Print results of completed exams |
| 80 | Laboratory | Lack of Standardized Process | Laboratory Exams | Mail exam results to clinical trial site |
| 81 | Laboratory Reception | CT Routine | Laboratory Exams | Collect exam request guides from research subjects |
| 82 | Laboratory Reception | Lack of Standardized Process | Laboratory Exams | Collect personal information about potential research subject and enter into laboratory information system |
| 83 | Laboratory Reception | Lack of Standardized Process | Laboratory Exams | Enter requested exams in laboratory information system |
| 84 | Laboratory Reception |  | Laboratory Exams | Direct research subject to specimen collections |
| 85 | Laboratory Reception | Lack of Standardized Process | Laboratory Exams | Adjourn encounter with research subject and clinical researcher coordinator |
| 86 | Laboratory Reception | CT Routine | Laboratory Exams | Collect exam request guides from research subjects |
| 87 | Laboratory Reception | Lack of Standardized Process | Laboratory Exams | Register requested exams in laboratory information system |
| 88 | Laboratory Reception | CT Routine | Laboratory Exams | Direct research subject to specimen collection |
| 89 | Laboratory Reception | CT Routine | Laboratory Exams | Direct research subject and research coordinator to nursing |
| 90 | Laboratory Technician | CT Routine | Laboratory Exams | Prepare research subject for specimen collection |
| 91 | Laboratory Technician | CT Routine | Laboratory Exams | Collect exam specimen |
| 92 | Laboratory Technician | CT Routine | Laboratory Exams | Release potential research subject |
| 93 | Laboratory Technician | CT Routine | Laboratory Exams | Send the specimen for clinical analysis |
| 94 | Laboratory Technician | CT Routine | Laboratory Exams | Direct research subject to laboratory reception |
| 95 | Laboratory Technician | CT Routine | Laboratory Exams | Prepare research subject for specimen collection |
| 96 | Laboratory Technician | CT Routine | Laboratory Exams | Collect exam specimen |
| 97 | Laboratory Technician | CT Routine | Laboratory Exams | Release research subject |
| 98 | Laboratory Technician | CT Routine | Laboratory Exams | Send specimen for clinical analysis |
| 99 | Laboratory Technician | CT Routine | Laboratory Exams | Direct research subject to laboratory reception |
| 100 | Research Coordinator | CT Routine | Laboratory Exams | Call laboratory and determine when exam is scheduled |
| 101 | Research Coordinator | Lack of Standardized Process | Laboratory Exams | Check exams in electronic medical record and schedule appointment |
| 102 | Research Coordinator | CT Routine | Laboratory Exams | Accompany the research subject to the laboratory for laboratory exams |
| 103 | Research Coordinator | CT Routine | Laboratory Exams | identify potential research subjects whose exams are pending |
| 104 | Research Coordinator | Lack of Standardized Process | Laboratory Exams | Obtain exam results through laboratory information system |
| 105 | Research Coordinator | Lack of Standardized Process | Laboratory Exams | Print laboratory results |
| 106 | Research Coordinator | Lack of Standardized Process | Laboratory Exams | Enter laboratory exam data in electronic medical record |
| 107 | Research Coordinator | Lack of Standardized Process | Laboratory Exams | Send printed exam results to PI for analysis |
| 108 | Research Coordinator | Lack of Standardized Process | Laboratory Exams | Print the laboratory exam requests from electronic medical record |
| 109 | Research Coordinator | CT Routine | Laboratory Exams | Accompany the research subject to the laboratory for laboratory exams |
| 110 | Research Coordinator | Lack of Standardized Process | Laboratory Exams | Check the laboratory system to verify exam results |
| 111 | Research Coordinator | Lack of Standardized Process | Laboratory Exams | Check e-mail software of clinical trial site |
| 112 | Research Coordinator | Lack of Standardized Process | Laboratory Exams | Print exam results |
| 113 | Research Coordinator | CT Routine | Laboratory Exams | Analyze exams results and follow the red path if the results are significant or the blue path if the results are within normal limits |
| 114 | Research Coordinator | CT Routine | Laboratory Exams | Ask the research subject to wait in the reception area |
| 115 | Nursing | CT Routine | Nursing Procedures | Greet research subject and lead him/her to exam room |
| 116 | Nursing | Lack of Standardized Process | Nursing Procedures | Record potential research subject's vital signs on nursing plan form. |
| 117 | Nursing | CT Routine | Nursing Procedures | Call pharmacy and request a prescription for research subject |
| 118 | Nursing | CT Routine | Nursing Procedures | Prepare research subject for infusion |
| 119 | Nursing | CT Routine | Nursing Procedures | Infuse drug according to clinical trial site standards, nursing plan and research protocol |
| 120 | Nursing | Lack of Standardized Process | Nursing Procedures | Record research subject's vital signs in nursing plan every pre-specified number of minutes according to protocol |
| 121 | Nursing | CT Routine | Nursing Procedures | Observe research subject for number of minutes specified in protocol |
| 122 | Nursing | CT Routine | Nursing Procedures | Direct research subject to research coordinator |
| 123 | Nursing | CT Routine | Nursing Procedures | Inject the research subject with the drug |
| 124 | Nursing | CT Routine | Nursing Procedures | Deliver oral drugs to research subject and instruct him/her on how to proceed |
| 125 | Nursing | Lack of Standardized Process | Nursing Procedures | Send nursing plan to research coordinator |
| 126 | Nursing | CT Routine | Nursing Procedures | Weight research subject and obtain vital signs |
| 127 | Nursing | Lack of Standardized Process | Nursing Procedures | Record data in the nusing plan |
| 128 | Nursing | Lack of Standardized Process | Nursing Procedures | Give nursing plan for research coordinator |
| 129 | Nursing | CT Routine | Nursing Procedures | Release research coordinator and research subject |
| 130 | Research Coordinator | CT Routine | Pre Signature Consent Form | Call the potential research subject and invite him/her to visit the clinical trial site |
| 131 | Clinical Trial Site Reception | CT Routine | Subject Reception | Greet potential research subject and ask for his/her identification |
| 132 | Clinical Trial Site Reception | CT Routine | Subject Reception | Ask research subject to wait in the reception area |
| 133 | Clinical Trial Site Reception | CT Routine | Subject Reception | Notify research coordinator that potential research subject has arrived |
| 134 | Clinical Trial Site Reception | CT Routine | Subject Reception | Greet the research subject and ask for his identification |
| 135 | Clinical Trial Site Reception | CT Routine | Subject Reception | Ask research subject to wait in the reception area |
| 136 | Clinical Trial Site Reception | CT Routine | Subject Reception | Notify the research coordinator that the research subject has arrived |
| 137 | Clinical Trial Site Reception | CT Routine | Subject Reception | Greet potential research subject and ask for his identificaiton |
| 138 | Clinical Trial Site Reception | CT Routine | Subject Reception | Notify the research coordinator that a potentail research subject has arrived |
| 139 | Clinical Trial Site Reception | CT Routine | Subject Reception | Ask potential research subject to wait in the reception area |
