# Supplementary material for: Workflow in Clinical Trial Sites & Its Association with Near Miss Events for Data Quality: Ethnographic, Workflow & Systems Simulation
Source: PLoS One. 2012 Jun 29;7(6):e39671. doi: 10.1371/journal.pone.0039671 (PMC3387261; doi:10.1371/journal.pone.0039671)

**S3 - UML - Activity Diagram for signature consent form and first visit**

**This file is available for download in:** [**http://goo.gl/QE5xY**](http://goo.gl/QE5xY)


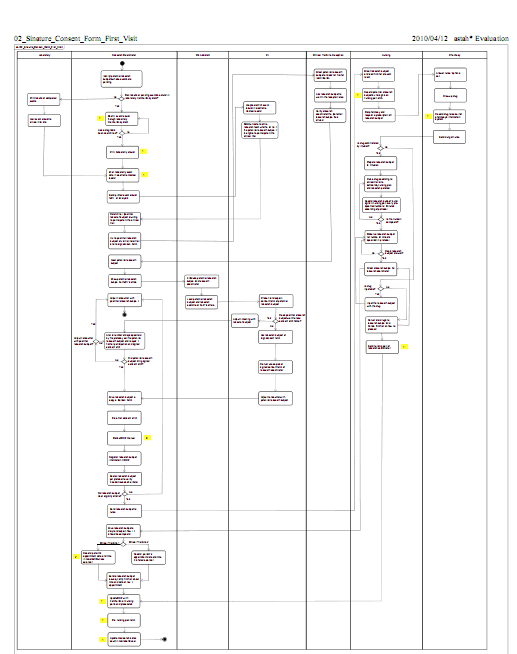

Supplement: Supporting Information S3 — UML - Activity Diagram for signature consent form and first visit. (DOC) [file pone.0039671.s003.doc]
