# Supplementary material for: Workflow in Clinical Trial Sites & Its Association with Near Miss Events for Data Quality: Ethnographic, Workflow & Systems Simulation
Source: PLoS One. 2012 Jun 29;7(6):e39671. doi: 10.1371/journal.pone.0039671 (PMC3387261; doi:10.1371/journal.pone.0039671)

**S4 - UML - Activity Diagram for next visits**

**This file is available for download in:** [**http://goo.gl/iUqRt**](http://goo.gl/iUqRt)


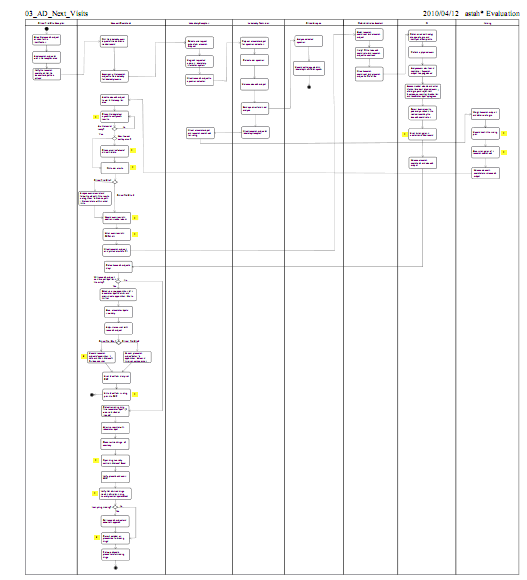

Supplement: Supporting Information S4 — UML - Activity Diagram for next visits. (DOC) [file pone.0039671.s004.doc]
