# Supplementary figures and images for: Workflow in Clinical Trial Sites & Its Association with Near Miss Events for Data Quality: Ethnographic, Workflow & Systems Simulation
Source: PLoS One. 2012 Jun 29;7(6):e39671. doi: 10.1371/journal.pone.0039671 (PMC3387261; doi:10.1371/journal.pone.0039671)

**S5 - Emergent themes main variables in Vensim simulation software**


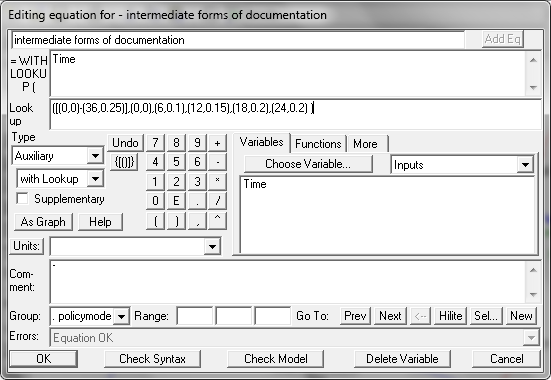


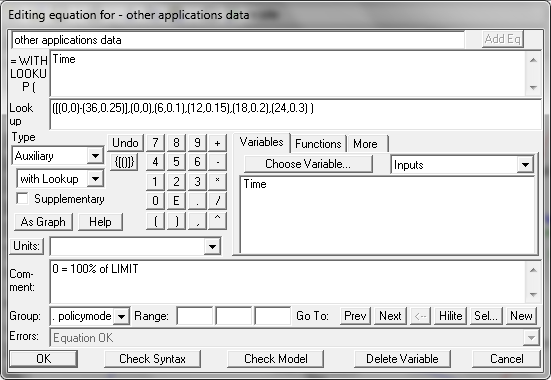


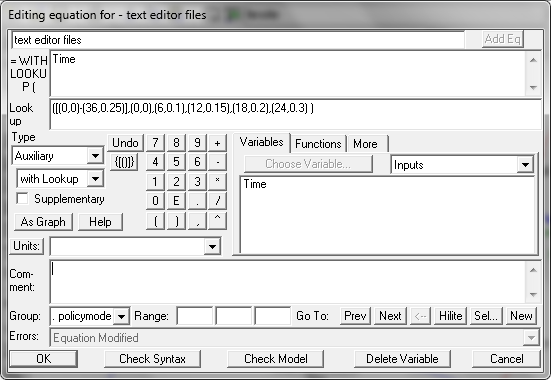


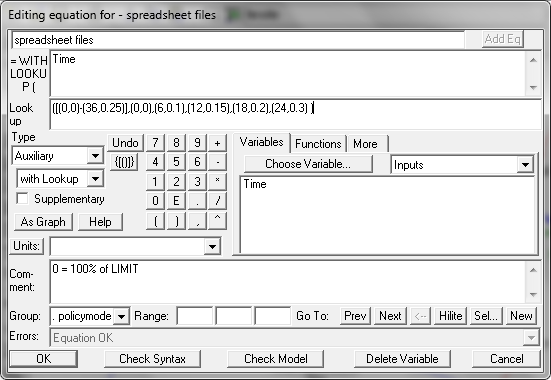


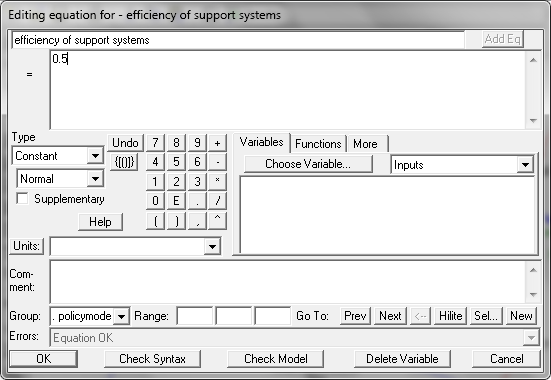

Supplement: Supporting Information S5 — Emergent themes main variables in Vensim simulation software. (DOC) [file pone.0039671.s005.doc]
