# Supplementary material for: Workflow in Clinical Trial Sites & Its Association with Near Miss Events for Data Quality: Ethnographic, Workflow & Systems Simulation
Source: PLoS One. 2012 Jun 29;7(6):e39671. doi: 10.1371/journal.pone.0039671 (PMC3387261; doi:10.1371/journal.pone.0039671)
Supplement: Supporting Information S6 — Workflow Terminology. (DOC) [file pone.0039671.s006.doc]

**S6 - Workflow Terminology**

**Therms**

We described below the terms used in the activity diagram

- **Activity:** an activity is a group of one or more actions that may execute as a result of a triggering event.
- **Activity diagram:** a diagram that shows activities and actions to describe workflows.
- **Actor:** an actor is an external entity of any form that interacts with the system. Actors may be physical devices, humans or information systems.
- **Astah Community:** is a free software to build UML diagrams
- **Decision:** a node in an activity diagram where a flow branches into multiple alternative flows
- **Event:** an occurrence that is of significance to the information system and may be included in a state machine.
- **Flow final node:** the final node in a flow within an activity diagram that terminates that flow but leaves other flows unaffected.Forces (of a pattern): the particular issues that must be addressed in resolving a problem.
- **Initial node:** the entry point to an activity diagram.
- **Task:** a specific activity or step in a project.
- **UML:** Unified Modeling Language - a standardized CT Routine-purpose modeling language used in object-oriented software engineering.

**Symbols**

The symbols below used in an activity diagram represents:

-
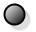
The begining of activity.
-
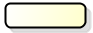
An activity to be done .
-
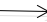
 The flow of activities.
-
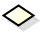
A decision to execute more one of the floes.
-
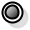
The end of activity.
